# Announcement of merger with journal of Clinical Labortory Automation

**DOI:** 10.1155/S1463924685000013

**Published:** 1985

**Authors:** Peter Stockwell


Journal of Automatic Chemistry, Volume 7, Number 1 (January–March 1985), pages 1–3

### IMPORTANT ANNOUNCEMENT

**We are pleased to announce that from 1 March 1985 the *Journal of Automatic Chemistry*, published by Taylor & Francis, and *The Journal of Clinical Laboratory Automation*, previously published by Appleton-Century-Crofts, are to merge to form one journal which will be published by Taylor & Francis.**

**This is the first issue of the newly merged journal. All previous subscribers to *The Journal of Clinical Laboratory Automation* are being sent this issue, subsequent issues will be mailed on payment of their renewal invoice.**

**In connection with this merger we are pleased to announce the appointment of a Managing Editor for North America – Margaret R. Stewart, 14 Shawnee Trail, Blacksburg, Virginia 24060, USA – to whom American and Canadian articles for publication should be addressed.**

### Editorial: Computers full circle

I have written before about analytical chemistry being a great follower of fashion. The recent spate of publicity on the so-called 'LIM' computer systems (see Dr Hornstein's article below has again had me reaching for my pen to issue my usual warning to the unwary purchaser of some sophisticated computer system which purports to fulfill his every need, but may just as easily throw the laboratory and all its staff into confusion and frantic panic.

In the early 1970s I was involved in the design, development and, later, successful implementation of a laboratory computer system. Despite a great deal of resistance because of its 'central facility', it proved reliable and economic. As it evolved there came the increasing requirement to cope with instruments with built-in microprocessors and to link other computers online. Less need was in fact found for initial data reduction but also there was an increasing requirement to share, or distribute, files of data or information and add such sophisticated computing facilities as high-quality plotters.

Whilst those of us putting forward the scheme were trying to rationalize the proposal with a distributive system we were in conflict with the advocates of the 'microprocessor for everyone and everyone independent of other users' bandwagon. Needless to say, few of these people had direct experience of operating a system. What seemed to appeal to this group was that the user could be independent, whereas centralization implied dependence. In reality each of us is dependent on others and on additional information. The need to communicate is vital in all areas, particularly in laboratories. Data presented in a form an analyst accepts will have no value to a customer who wants an answer to his own questions and also some clear idea of the reliability of that data.

The distributed approach that was evolved in response to large laboratories' needs has a remarkable similarity to the various LIM systems currently being promoted. However, it does have a distinct and important difference. The instruments included within the distribution net were not specifically from one company. Also the reporting and archiving software requirements were developed specifically for individual's requirements in a particular laboratory.

Specifying the precise needs of any laboratory computer system are difficult without a clear understanding of the resources of hardware and software available. It is impossible without a detailed overview of what the laboratory requirements actually are. On the latter it is vital not to be clouded by those facilities a laboratory actually offers at present—the specification process must look beyond this aspect to the underlying requirements. A recent survey on laboratory data processing in the UK showed clearly the level of interest in implementing systems of this nature and equally clearly the lack of any real penetration at present. With increased pressure to improve productivity with reductions in manpower there will be continued pressure on management to install LIM-type systems. Given proper attention to a detailed specification the successful manager can look forward to a successful period. The unwary may invest in a very expensive and continued headache.

**Peter Stockwell**

